# The association between dietary diversity and hearing loss: results from a nationwide survey

**DOI:** 10.3389/fnut.2025.1629685

**Published:** 2025-09-08

**Authors:** Qiaoqiao Du, Haizhen Yang, Hua Zhang, Yao Yao, Zhongmin Wen, Xiaojun Zhu, Xiangyang Gao

**Affiliations:** ^1^Health Management Center, The Second Affiliated Hospital of Soochow University, Suzhou, China; ^2^Research Center of Clinical Epidemiology, Peking University Third Hospital, Beijing, China; ^3^China Center for Health Development Studies, Peking University, Beijing, China; ^4^Health Management Center of The Second Affiliated Hospital of Luohe Medical College, Luohe, China

**Keywords:** dietary diversity, older adults, hearing loss, plant-based diet, CLHLS

## Abstract

**Background:**

Hearing loss is a leading cause of disability among older adults. Previous studies have suggested that dietary factors may play a role in preventing hearing loss. However, findings remain inconsistent, and longitudinal evidence is limited. Based on a national sample, this study aims to explore the association between the different dietary diversity score (DDS) and hearing loss in the older adult Chinese population.

**Methods:**

We analyzed longitudinal data from the Chinese Longitudinal Healthy Longevity Survey (CLHLS) spanning 2011 to 2018. The study recruited participants aged 60 years and older from the 2011 and 2014 CLHLS waves, with follow-up assessments in 2014 and 2018. Hearing loss was assessed through a questionnaire, while dietary diversity was evaluated using four DDS indicators: total diet, animal-based diet, protein-based diet, and plant-based diet. Cox regression models were employed to examine the relationship between various DDS categories and hearing loss, with adjustments for confounding factors. Restricted cubic spline (RCS) regression was employed to explore the relationship between DDS and hearing loss. Subgroup analyses and sensitivity analyses were performed to further validate the findings.

**Results:**

The cohort study included 3,839 older adults (mean age 79.1 ± 9.3 years) without hearing loss at baseline, comprising 1874 males (48.8%) and 1965 females (51.2%). The mean follow-up duration was 4.2 years (SD 1.9). The incidence of hearing loss was 47.6% (1827/3839). After adjusting for confounding factors, higher plant-based DDS (HR = 0.783; 95% CI: 0.637–0.962, *P*-trend = 0.027) was associated with a reduced risk of hearing loss. The RCS analysis indicated a negative linear association between plant-based DDS and hearing loss (*p* for linearity = 0.014). Sensitivity analysis further verified the robustness of the above findings. Similar results were observed in adults aged ≥80 years, males, and individuals without hypertension, diabetes, or heart disease.

**Conclusion:**

Dietary diversity in plant-based diet serves as a protective factor against hearing loss in the older adult population. Adopting a diversified plant-based diet may help reduce the risk of hearing loss among older adults.

## 1 Introduction

Hearing plays a crucial role in social communication and cognitive functioning. With global life expectancy rising, age-related hearing loss has emerged as a major public health challenge, ranking among the top causes of disability in older adult populations worldwide (1, 2). According to the 2019 Global Burden of Disease (GBD) Study, hearing loss affects over 1.57 billion individuals globally, and is potentially reaching 2.31 billion people by 2050 (3, 4). China has experienced a particularly pronounced increase in hearing loss prevalence with population aging. Age-related hearing loss (ARHL) constitutes the primary etiology of hearing disability nationwide. Epidemiological data show that 68.2% of all hearing loss cases in China are concentrated among middle-aged and older adult aged 50 and above (3). Among octogenarians with hearing loss, approximately 50% present with moderate-to-profound auditory loss (3).

The consequences of hearing loss extend beyond sensory loss, significantly impacting quality of life, mental health, social relationships, and cognitive function (1, 5). Studies have demonstrated that hearing loss adversely impacts the communication and social engagement of older adults, which may subsequently increase the risk of cognitive decline, dementia, depression, anxiety, and loneliness (6–10). The etiology of hearing loss is multifactorial, involving genetic predisposition, environmental exposures, oxidative stress, and chronic inflammation (5, 11–15). Recent research has highlighted the potential role of modifiable lifestyle factors, particularly diet, in hearing loss prevention (16–19). Despite the complexities involved in elucidating the relationships between nutrients, overall diet, and auditory function, the literature does include a number of studies that provide evidence (17). Specific dietary nutrients have demonstrated dual effects on auditory health, with some components showing protective effects while others may increase hearing loss risk (20–23). Previous studies have suggested that increasing consumption of vegetables, fruits, and cereal fiber has been associated with reduced hearing loss susceptibility (24). Supplementation with omega-3 polyunsaturated fatty acids (PUFAs), individual antioxidant, and mineral (calcium and magnesium) are associated with a decreased risk of hearing loss (19, 21, 25–27). Conversely, a high-fat diet, high intake of carbohydrates and sugars appears to correlate positively with audio-vestibular dysfunction (1, 18, 19, 28–30).

Studying individual nutrients or foods may offer an incomplete understanding of the relationship between diet and hearing loss, as diets are typically composed of combinations of foods and involve complex interactions among nutrients in our daily consumption. This highlights the need for comprehensive dietary analysis to better understand the relationship between nutrition and auditory health. Research evidence suggested that better diet quality and dietary patterns were significantly associated with a reduced risk of hearing loss (18, 31–33). An inflammatory diet may increase the risk of developing sensorineural hearing loss (34). Healthy dietary patterns, particularly the DASH diet, were associated with a reduced risk of hearing loss and a slower cognitive decline following hearing loss (7). The Mediterranean diet and plant-based diets have been shown to contribute to the prevention of auditory vestibular dysfunction (30, 35). However, no significant associations were observed between dietary intake and the prevalence of hearing loss in the Blue Mountains Hearing Study (Australia) (33, 36).

Dietary diversity score (DDS) has been recognized as a practical and reliable indicator of overall diet quality, capturing the variety of foods consumed over a specified period (37). DDS has been established as a reliable and practical tool in large-scale epidemiological research, offering multiple methodological advantages, including its utility as a robust indicator of nutrient adequacy, its capacity for cross-cultural dietary comparisons, and its cost-effectiveness as a dietary assessment instrument (37–39). Previous studies have established associations between DDS and various health outcomes, including reduced risks of cardiovascular diseases, metabolic syndrome and improved cognitive performance (39–42). In developing countries, where dietary patterns often lack diversity, promoting dietary variety has become an important public health strategy (39).

Despite the growing interest in the associations between diet and hearing loss, many previous studies have reported inconsistent results and a limited number of longitudinal investigations. Furthermore, the majority of studies have been carried out in developed countries, with limited evidence from developing nations, particularly in China, which has the world’s largest older population. This study aims to address these gaps by investigating the association between DDS and hearing loss prevalence among Chinese older adults. Using nationwide longitudinal data, we seek to provide evidence-based insights for developing dietary guidelines to prevent hearing loss in aging populations.

## 2 Materials and methods

### 2.1 Study population

**Figure 1:**
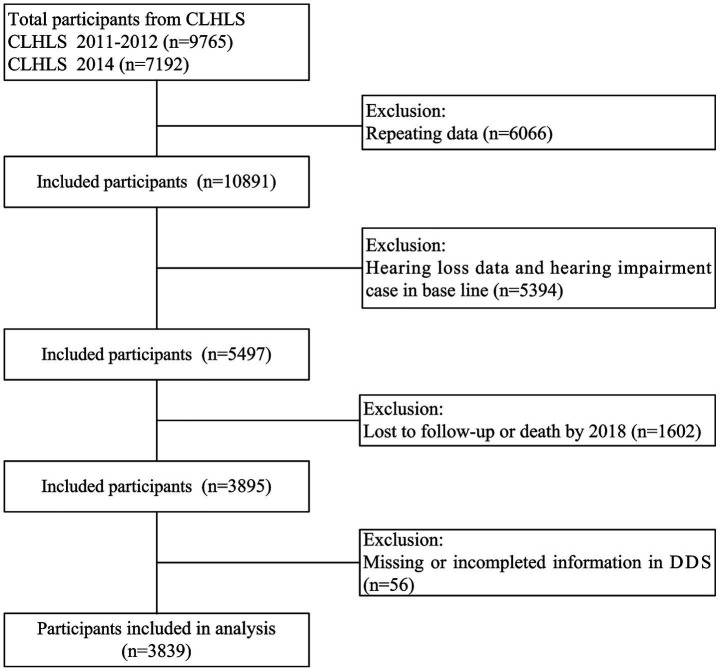
Flowchart of participants selection.

Data from the Chinese longitudinal healthy longevity survey (CLHLS) were utilized in this study. The CLHLS was conducted in 23 of the 31 provinces in mainland China employing a multistage, stratified cluster sampling design. The details of the sampling strategy have been thoroughly discussed in our prior studies (43, 44). This study selected the survey data of CLHLS from 2011 to 2018, with baseline participants recruited in 2011–2012 (*n* = 9,765) and in 2014 (*n* = 7,192). After excluding duplicate records (*n* = 6,066), 10,891 individuals were retained for initial analysis. We further excluded participants with incomplete hearing data or baseline hearing loss (*n* = 5,394), leaving 5,497 eligible individuals. Subsequent attrition due to loss to follow-up or death by 2018 (*n* = 1,602) reduced the sample to 3,895. Finally, after excluding cases with incomplete dietary diversity score (DDS) information (*n* = 56), 3,839 participants were included in the final analysis (Figure 1). Informed consent was obtained from all participants and/or their legal guardians, and the study received approval from the Ethics Committee of Peking University (IRB00001052-13074).

### 2.2 Assessment of dietary diversity

DDS was developed in alignment with the Chinese Dietary Guidelines, which provide recommendations for the consumption of 10 primary food groups (38, 42). Baseline DDS assessment was conducted utilizing a validated food frequency questionnaire encompassing 10 core food categories: cereals, vegetables, fruits, legumes, nuts, meat, eggs, fish, dairy products, and fungi. The scoring criteria were defined as follows: For cereals, vegetables, and fruits, a score of one point per category was assigned for regular consumption (“daily,” “almost daily,” or “frequently”); otherwise (“occasionally,” “rarely,” or “never”), 0 points were assigned. For fungi, legumes, nuts, meat, eggs, fish, and dairy products, one point per category was given for consumption (“almost daily” or “not daily but at least weekly”); otherwise (“less than weekly but at least monthly,” “less than monthly, sometimes,” “rarely,” or “never”), 0 points were assigned. The DDS of total diet for each participant ranges from 0 to 10. Based on previous research, we further evaluated different dietary diversities through the animal food group, plant food group, and protein food group (45, 46). The animal-based DDS, derived from four primary sources (meat, fish, eggs, and dairy products), ranged from 0 to 4 points based on consumption frequency. Similarly, the plant-based DDS, incorporating six food sources (cereals, vegetables, fruits, legumes, nuts, and fungi), was calculated on a 0 to 6-point scale. Protein-rich food diversity was assessed across six sources (legumes, nuts, meat, eggs, fish, and dairy products), with the protein-based DDS ranging from 0 to 6 points based on consumption frequency.

### 2.3 Assessment of hearing loss

Hearing loss was evaluated through a dual assessment approach incorporating investigator observations and participant self-reports, utilizing a questionnaire that assessed auditory function through two primary questions. Questions 1 “Can you hear the interviewer’s questions clearly?” with response options: (1) yes, without requiring hearing aids; (2) yes, but requiring hearing aids; (3) partially, requiring hearing aids; and (4) no, cannot hear clearly. Questions 2 “Do you experience hearing difficulties?” with binary response options (Yes/No). Participants were classified as having hearing loss if they selected options (2), (3), or (4) for the first question and/or responded affirmatively (“Yes”) to the second question. All other responses were categorized as indicating normal hearing. Given that the specific onset time of hearing loss was not recorded in the CLHLS, this study estimated the occurrence of hearing loss under various scenarios. For participants who did not report hearing loss at their most recent follow-up, the event timing was determined as the difference between the year of the last survey and the baseline year. For those who did develop hearing loss, the timing was based on the difference between the earliest survey year of hearing loss onset and the baseline year.

### 2.4 Covariates

To enhance the reliability of our findings, we controlled for an extensive range of potential confounders. Demographic variables included gender (male/female), age group (60–79 years/≥80 years), socioeconomic factors (educational attainment [0, 1–6, and ≥7 years], marital status [married, unmarried, divorced, or widowed], pre-retirement occupation [agricultural/non-agricultural], and household income [<100,000/≥100,000 yuan]), lifestyle characteristics (sleep quality [good/general or poor], current smoking [yes/no], current consuming alcohol [yes/no], and current physical activities [yes/no]), chronic diseases (yes/no), and body mass index (BMI). Weight and height were obtained using standardized anthropometric protocols. Body mass index (BMI) was calculated as weight in kilograms divided by the square of height in meters (kg/m^2^). The chronic diseases primarily encompassed hypertension, diabetes and heart disease.

### 2.5 Statistical analysis

The data were presented as mean ± standard deviation (SD) for continuous variables and as percentages for categorical variables. Group comparisons were performed using Chi-square tests for qualitative variables and independent *t*-tests for quantitative variables. Missing covariates were addressed through multiple imputation methods. The hazard ratios (HRs) for hearing loss associated with DDS, analyzed both as continuous and quartile categorical variables, were estimated using cox proportional hazards models. Four sequential models were constructed: Model 1 was unadjusted. Model 2 was adjusted to account for demographic factors, including gender, age, household income, education level, marital status, and pre-retirement occupation. Model 3 additionally adjusted for lifestyle factors, including physical activity, smoking, alcohol consumption, and sleep status, and Model 4 further adjusted for clinical factors (BMI, hypertension, diabetes, and cardiovascular disease). To assess the robustness of our findings, sensitivity analyses were conducted by excluding participants who were unable to respond due to hearing loss and those with pre-existing hearing problems before age 40. Stratified analyses were performed based on gender (male/female), age group (60–79 years/≥80 years), chronic diseases, and interactions between strata were examined. A restricted cubic spline analysis was employed to investigate the dose–response relationship between DDS and hearing loss. Statistical analyses were performed using R software version 4.4.6. A two-tailed *p*-value < 0.05 was considered statistically significant.

## 3 Results

### 3.1 The characteristics of study participants

The study population comprised 1,965 females (51.2%), 1,924 married individuals (50.3%), 1,727 urban residents (45.0%), and 3,001 manual laborers (80.2%), with a mean age of 79.84 ± 9.9 years, stratified into 2071 participants aged 60–79 years and 1,768 participants aged ≥80 years. Educational attainment distribution revealed 1859 illiterate participants (48.6%), 1,400 with primary education (36.6%), and 569 with junior high school or higher education (14.9%). Participants were categorized into quartiles based on baseline DDS levels, with comparative analyses demonstrating statistically significant differences (*P* < 0.05) across all demographic characteristics except smoking and consuming alcohol. The mean follow-up duration was 4.2 years (SD 1.9). Longitudinal follow-up revealed an overall hearing loss incidence of 47.6% (1827/3839), with statistically significant interquartile differences (*P* = 0.023), showing the lowest incidence rate in the highest DDS quartile group (Table 1).

**Table 1:** Baseline characteristics of all participants by quartiles of DDS.

Characteristics	Total, *n* (%)	Quartiles of DDS, *n* (%)				*χ^2^*	*P*
		Q1(0–4] (*n* = 1,420)	Q2(4–5] (*n* = 737)	Q3(5–6] (*n* = 752)	Q4(6–10] (*n* = 930)		
Gender						14.691	0.002
Male	1874(48.8)	642(45.2)	356(48.3)	394(52.4)	482(51.8)		
Female	1965(51.2)	778(54.8)	381(51.7)	358(47.6)	448(48.2)		
Age group, years						40.982	<0.001
60–79	2071(53.9)	706(50.3)	371(50.3)	414(55.1)	580(62.4)		
≥80	1768(46.1)	714(49.7)	366(49.7)	338(44.9)	350(37.6)		
Residence						181.171	<0.001
City, Town	1727(45.0)	475(33.5)	315(42.7)	368(48.9)	569(61.2)		
Rural	2,112(55.0)	945(66.5)	422(57.3)	384(51.1)	361(38.8)		
Marital status						61.377	<0.001
Married	1924(50.3)	617(43.5)	359(48.8)	396(52.8)	552(59.7)		
Unmarried, divorced, or widowed	1903(49.7)	800(56.5)	377(51.2)	354(47.2)	372(40.3)		
Education level, years						199.162	<0.001
0	1859(48.6)	813(57.5)	383(52.1)	345(45.9)	318(34.3)		
1–6	1,400(36.6)	469(33.2)	265(36.1)	305(40.6)	361(38.9)		
7+	569(14.9)	131(9.3)	87(2.3)	102(13.6)	249(26.8)		
Pre-retirement occupation						116.961	<0.001
Peasant	3,001(80.2)	1,199(86.9)	597(82.9)	578(79.2)	627(68.8)		
No-peasant	740(19.8)	181(13.1)	123(17.1)	152(20.8)	284(31.2)		
Household income, *yuan*						29.319	<0.001
≥100,000	148(3.9)	35(2.5)	21(2.9)	30(4.1)	62(6.8)		
<100,000	3,612(96.1)	1,351(97.5)	704(97.1)	709(95.9)	848(93.2)		
Sleep status						78.957	<0.001
Good	2,455(64.0)	788(55.5)	494(67.0)	497(66.1)	676(72.7)		
General, Poor	1,383(36.0)	631(44.5)	243(33.0)	255(33.9)	254(27.3)		
BMI (kg/m^2^)						86.976	<0.001
<18.5	686(18.4)	324(23.6)	132(18.6)	118(16.1)	112(12.3)		
18.5 ~ 24.0	2012(54.4)	754(54.9)	375(52.9)	414(56.6)	469(51.4)		
≥24.0	1,028(27.6)	295(21.5)	202(28.5)	200(27.3)	331(36.3)		
Current Smoking	764(19.9)	273(19.2)	160(21.8)	162(21.5)	169(18.2)	4.934	0.177
Current consuming alcohol	760(19.8)	263(18.5)	162(22.0)	143(19.0)	192(20.6)	4.380	0.223
Current physical activities	1,622(42.3)	483(34.0)	297(40.3)	334(44.4)	508(54.6)	100.428	<0.001
Hypertension	1,176(31.7)	417(30.8)	215(30.2)	217(29.6)	327(36.1)	10.817	0.013
Diabetes	192(5.2)	47(3.5)	33(4.7)	41(5.6)	71(7.9)	21.965	<0.001
Heart disease	446(12.1)	130(9.6)	72(10.3)	95(13.1)	149(16.6)	27.978	<0.001
Hearing loss	1827(47.6)	689(48.5)	379(51.4)	347(46.1)	412(44.3)	9.503	0.023

DDS, dietary diversity score; BMI, body mass index. Q1–Q4: Lowest quartile to the highest quartile.

### 3.2 DDS and hearing loss

**Figure 2:**
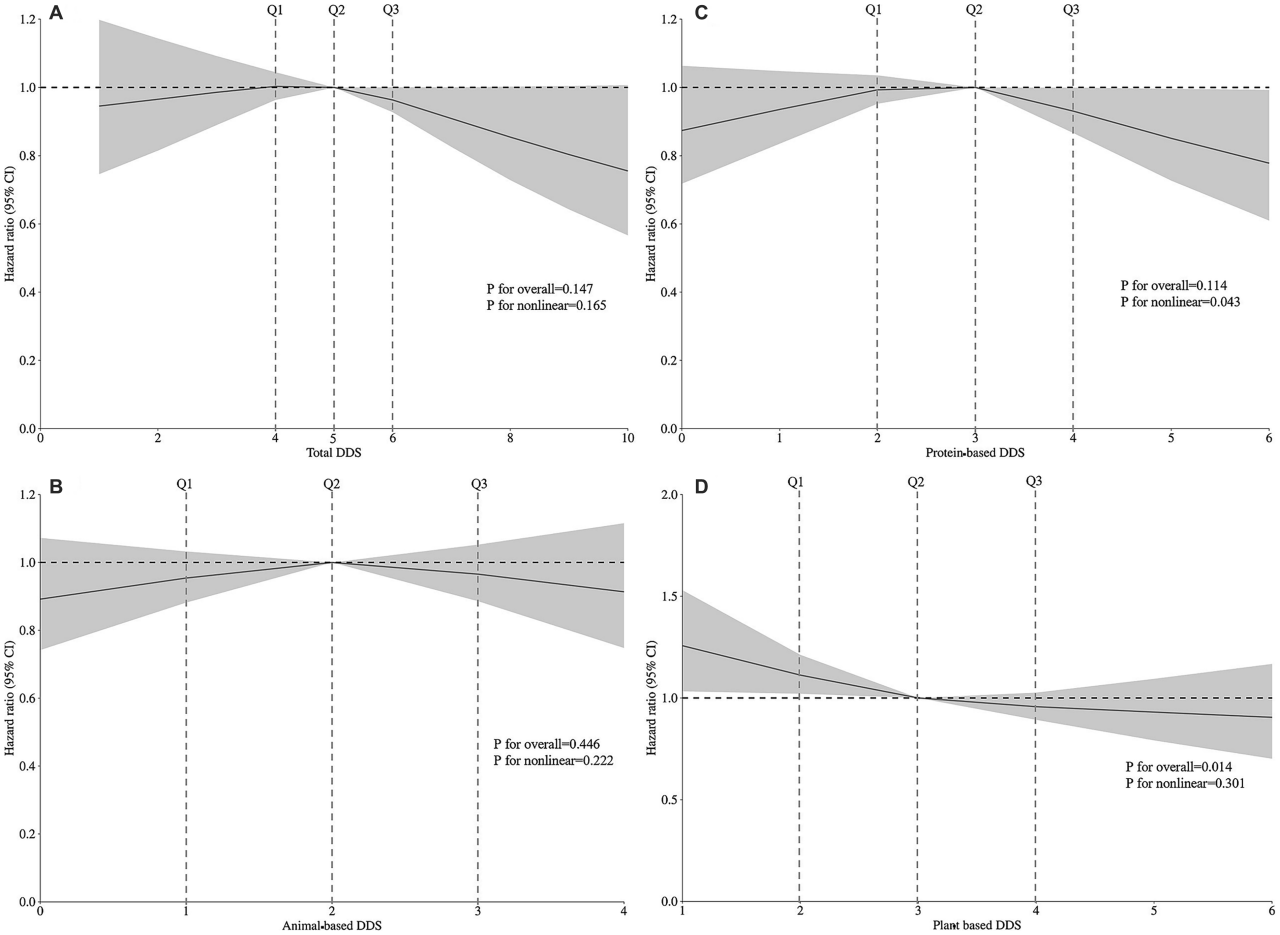
Restricted cubic spline analysis showing the dose–response relationships between **(A)** total DDS, **(B)** animal-based DDS, **(C)** protein-based DDS, **(D)** plant-based DDS, and the risk of hearing loss based on Model 4. Model 4 was adjusted for gender, age, household income, education level, marital status, pre-retirement occupation, physical activities, current smoking, current consuming alcohol, sleep quality, BMI, hypertension, diabetes, and heart disease. Total DDS ranges from 0 to 10; Animal-based DDS ranges from 0 to 4; Protein-based DDS ranges from 0 to 6; Plant-based DDS ranges from 0 to 6.

In Table 2, Cox regression models were employed to evaluate the associations between total DDS and DDS from different food sources with hearing loss risk. In Model 1, higher DDS (4th vs. 1st quartile) was associated with reduced hearing loss incidence. Total diet (HR = 0.834, 95% CI: 0.738–0.942), animal-based diet (HR = 0.752, 95% CI: 0.601–0.941), protein-based diet (HR = 0.773, 95% CI: 0.649–0.921), and plant-based diet (HR = 0.665, 95% CI: 0.555–0.797). Trend analyses revealed significant dose–response relationships for total diet (*P*-trend = 0.002), protein-based diet (*P*-trend = 0.007), and plant-based diet (*P*-trend<0.001). In Model 2 adjusted for gender, age, residence, annual household income, education level, marital status, and pre-retirement occupation, these associations persisted. Specifically, plant-based diet (HR = 0.749, 95% CI: 0.616–0.910) remained inversely associated with hearing loss in the highest quartile. Model 3 additionally adjusted for physical activity, smoking, alcohol consumption, and sleep duration maintained significant inverse associations for plant-based diet (HR = 0.783, 95% CI: 0.644–0.953). In Model 4 with further adjustment for hypertension, diabetes, cardiovascular disease, and BMI, the protective associations remained for plant-based diet (HR = 0.783, 95% CI: 0.637–0.962), with trend analyses remaining significant (*P*-trend = 0.027). Continuous plant-based diet DDS analysis also demonstrated significant associations. A restricted cubic spline analysis was employed to examine the dose–response relationship between DDS and hearing loss. After adjusting for covariates, we observed that only plant-based diet DDS (*P* for linearity = 0.014) showed a linear relationship with hearing loss (Figure 2).

**Table 2:** Hazard ratio (HR) of hearing loss by quartiles of DDS among whole sample.

DDS	Model 1	Model 2	Model 3	Model 4
Total (continuous)	0.952(0.928,0.977)***	0.972(0.945,1.000)	0.984(0.957,1.013)	0.980(0.951,1.011)
Q1(0,4]	Reference	Reference	Reference	Reference
Q2(4,5]	1.052(0.928,1.192)	1.071(0.942,1.217)	1.092(0.960,1.242)	1.117(0.975,1.280)
Q3(5,6]	0.910(0.800,1.036)	0.945(0.826,1.080)	0.966(0.845,1.105)	0.956(0.830,1.102)
Q4(6,10]	0.834(0.738,0.942)**	0.915(0.802,1.046)	0.954(0.834,1.091)	0.953(0.826,1.098)
*P-*trend	0.002	0.001	0.352	0.324
Animal-based (continuous)	0.973(0.932,1.016)	1.000(0.954,1.047)	1.012(0.966,1.060)	1.011(0.963,1.062)
Q1(0,1]	Reference	Reference	Reference	Reference
Q2(1,2]	0.975(0.868,1.096)	1.001(0.887,1.129)	1.022(0.906,1.154)	1.045(0.919,1.187)
Q3(2,3]	1.001(0.897,1.124)	1.048(0.931,1.180)	1.071(0.950,1.206)	1.079(0.952,1.224)
Q4(3,4]	0.752(0.601,0.941)**	0.859(0.676,1.092)	0.906(0.712,1.153)	0.881(0.685,1.133)
*P*-trend	0.210	0.574	0.635	0.695
Protein-based (continuous)	0.962(0.932,0.994)*	0.984(0.950,1.019)	0.993(0.958,1.029)	0.991(0.954,1.029)
Q1(0,2]	Reference	Reference	Reference	Reference
Q2(2,3]	0.965(0.857,1.085)	0.986(0.873,1.113)	0.999(0.885,1.128)	1.016(0.894,1.154)
Q3(3,4]	0.926(0.825,1.040)	0.956(0.847,1.079)	0.974(0.862,1.101)	0.974(0.857,1.107)
Q4(4,6]	0.773(0.649,0.921)**	0.866(0.717,1.046)	0.899(0.744,1.086)	0.873(0.714,1.068)
*P-*trend	0.007	0.046	0.344	0.288
Plant-based (continuous)	0.894(0.856,0.933)***	0.927(0.884,0.971)**	0.939(0.895,0.985)*	0.936(0.890,0.984)*
Q1(0,2]	Reference	Reference	Reference	Reference
Q2(2,3]	0.837(0.748,0.937)**	0.869(0.773,0.976)*	0.884(0.787,0.994)*	0.878(0.776,0.993)*
Q3(3,4]	0.831(0.737,0.938)**	0.895(0.788,1.016)	0.925(0.814,1.052)	0.906(0.791,1.038)
Q4(4,6]	0.665(0.555,0.797)***	0.749(0.616,0.910)*	0.783(0.644,0.953)*	0.783(0.637,0.962)*
*P-*trend	**<0.001**	**0.001**	**0.032**	**0.027**

Model 1 included DDS as the sole variable; Model 2 further controlled for gender, age, household income, education level, marital status and pre-retirement occupation; Model 3 additionally controlled for current physical activities, current smoking, current consuming alcohol, sleep quality; Model 4 added difficulty in BMI, hypertension, diabetes and heart disease; DDS, dietary diversity score; BMI, body mass index; Q1–Q4: Lowest quartile to the highest quartile; Q1 as the reference group; The bold values represent the statistically significant hazard ratios; *P < 0.05, **P < 0.01, ***P < 0.001.

### 3.3 Subgroup analyses

**Figure 3:**
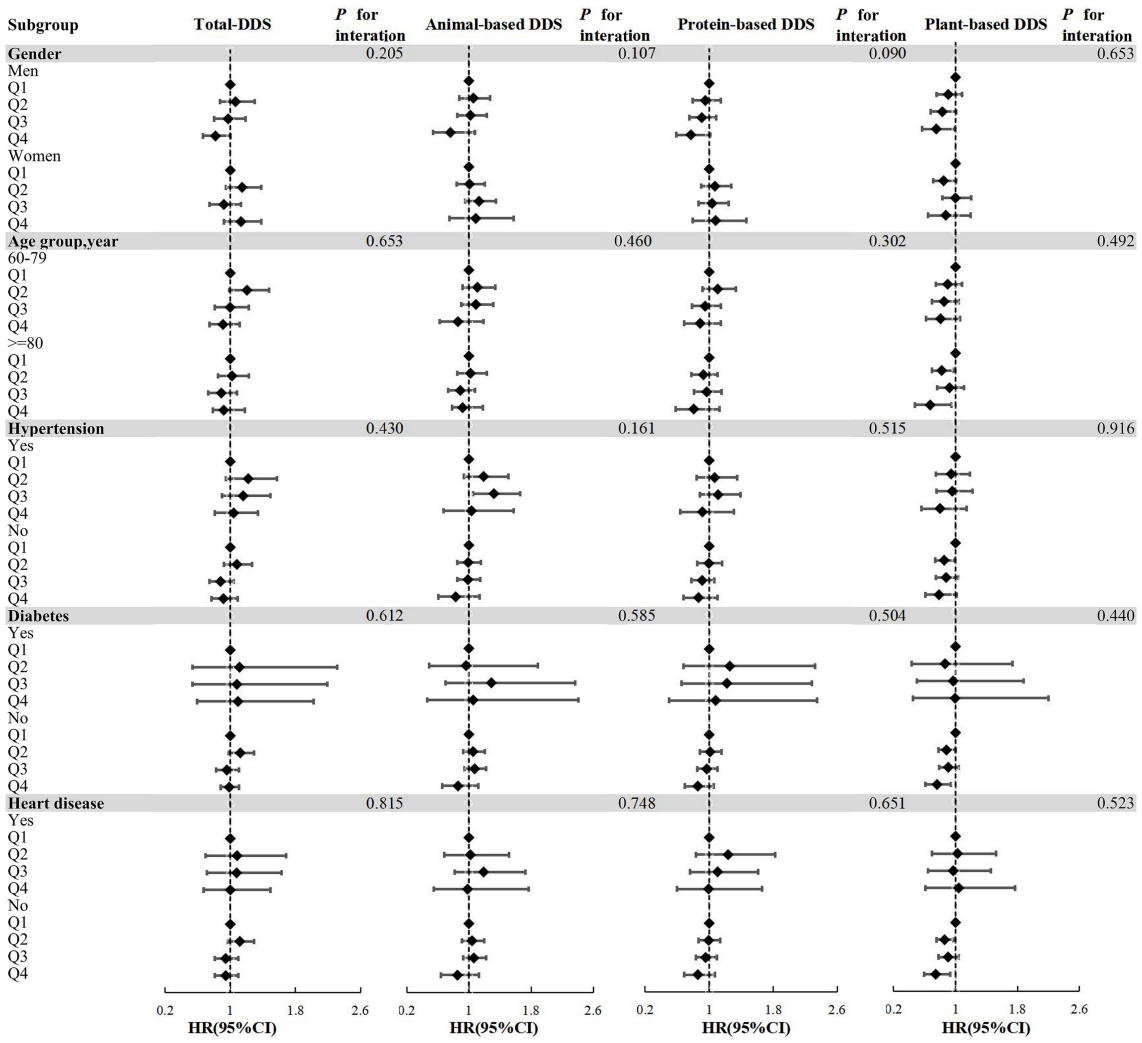
Forest plots of the associations between DDS and risk of hearing loss in subgroups based on Model 4. Subgroups according to gender, age group, hypertension, diabetes, heart disease; Model 4 adjusted for gender, age, household income, education level, marital status, pre-retirement occupation, physical activities, current smoking, current consuming alcohol, sleep quality, BMI, hypertension, diabetes, and heart disease; DDS, dietary diversity score; Q1-Q4: Lowest quartile to the highest quartile; Q1 as the reference group; all *P* for interaction >0.05.

Subgroup analysis conducted with Model 4 identified associations between plant-based diets and the risk of hearing loss across different gender, age and chronic diseases. Among males, plant-based DDS exhibited a protective effect (HR = 0.751, 95% CI: 0.567–0.995) in the highest quartile, supported by significant trend analysis (*P*-trend = 0.019). In the cohort aged ≥80 years, plant-based diets were associated with a reduced risk of hearing loss (HR = 0.672, 95% CI: 0.477–0.948). However, trend analysis did not achieve statistical significance (*P* = 0.064). Among people without hypertension, compared with the lowest quartile, the second quartile of plant-based DDS had a significant protective effect (HR = 0.853, 95% CI: 0.736–0.987; *P*-trend = 0.043). Among individuals without diabetes, compared with the lowest quartile, the highest quartile of plant-based DDS showed a significant protective effect (HR = 0.761, 95% CI: 0.614–0.944; *P*-trend = 0.021). Among individuals without heart disease, compared with the lowest quartile, the HR (95% CI) for the second quartile and the highest quartile were 0.858 (0.753–0.977) and 0.743 (0.592–0.933), respectively. The trend analysis indicates significance (*P*-trend = 0.019). No statistically significant associations were observed among females or individuals aged 60–79 years or with chronic diseases (Supplementary Table 1). Figure 3 presents the results of the subgroup analyses, indicating that no significant interactions were detected when comparing the classes (*P* for interaction > 0.05).

### 3.4 Sensitivity analyses

Sensitivity analysis across various DDS categories yielded consistent results with Model 4, demonstrating stable associations between all DDS measures (total diet, animal-based diet, protein-based diet, and plant-based diet) and hearing loss risk, maintaining alignment with the findings presented in Table 2, even after the exclusion of participants who were unable to respond to questions due to hearing loss and those who developed hearing problems before age 40 (n = 74), as detailed in Table 3.

**Table 3:** Sensitivity analysis of DDS with hearing loss based on Model4.

DDS	Total	Animal-based	Protein-based	Plant-based
Continuous	0.980(0.951,1.011)	1.010(0.961,1.061)	0.990(0.953,1.029)	0.937(0.891,0.986)*
Q1	Reference	Reference	Reference	Reference
Q2	1.107(0.964,1.271)	1.040(0.913,1.184)	1.017(0.893,1.158)	0.885(0.781,1.003)
Q3	0.957(0.829,1.105)	1.076(0.948,1.223)	0.973(0.854,1.108)	0.912(0.794,1.046)
Q4	0.956(0.828,1.103)	0.882(0.686,1.135)	0.877(0.717,1.073)	0.789(0.642,0.971)*
*P* trend	0.355	0.736	0.294	0.036

The results were based on Model 4 controlling for gender, age, household income, education level, marital status, pre-retirement occupation, physical activities, current smoking, current consuming alcohol, sleep quality, BMI, hypertension, diabetes, and heart disease; DDS, dietary diversity score; Q1–Q4: Lowest quartile to the highest quartile; Q1 as the reference group; *P < 0.05.

## 4 Discussion

This study evaluated associations of DDS with hearing loss and revealed a potential protective effect of DDS. We utilized a longitudinal dataset comprising one of the most extensive collections of the oldest-old populations in China and stratified by gender and age. Our study findings indicate that higher total diet DDS, particularly in the highest quartile, is associated with the lowest incidence of hearing loss among Chinese older adults. Specifically, plant-based DDS demonstrated a significant negative correlation with hearing loss. As the quartiles of DDS increased, the risk of hearing loss decreased. Sensitivity analyses confirmed the robustness of these associations, particularly for plant-based diets. Subgroup analyses further revealed that these associations were more pronounced in men. However, no significant associations were observed between total diet, animal-based diet, protein-based diet and hearing loss.

Previous studies have demonstrated that diversified, well-balanced, and nutritionally dense dietary patterns hold significant potential to decelerate the aging process and promote healthy aging via multiple underlying mechanisms (24, 38). The adherence to healthy eating patterns has been shown to be associated with numerous positive health outcomes (47). Multiple studies have shown that a diet high in variety, especially one rich in antioxidants, dietary fiber, Omega-3 fatty acids, niacin, retinol and folic acid, is associated with a reduced risk of hearing loss (5, 18, 19, 26). According to the National Health and Nutrition Examination Study (NHANES), adherence to a Mediterranean-style diet, characterized by high diversity and richness in fruits, vegetables, whole grains, and olive oil, was significantly associated with a reduced likelihood of high-frequency hearing loss among individuals aged 50 years or older (42). Better diet quality, as evaluated by indices such as the Alternate Mediterranean Diet (AMED), Dietary Approaches to Stop Hypertension (DASH), and the Alternative Healthy Eating Index-2010 (AHEI-2010), was associated with a reduced risk of self-reported hearing loss in female population (31). A cross-sectional study has shown that higher dietary diversity is associated with better auditory sustained attention (39). Dietary diversity may reduce oxidative damage in cochlea through the synergistic action of multiple nutrients. DDS is a quantitative tool to assess dietary variety, promoting comprehensive and balanced nutrition. By emphasizing overall dietary patterns rather than isolated nutrients or foods, it helps mitigate malnutrition risks and diet-related diseases linked to monotonous eating habits. Additionally, the DDS ensures adequate intake of essential macronutrients and micronutrients through diversified food choices.

The observed associations between plant-based dietary diversity and hearing loss may potentially be attributed to multiple underlying mechanisms. Oxidative stress and systemic inflammation are key contributors to age-related hearing loss (48). A diverse diet, particularly one rich in plant-based foods, may protect hearing function through antioxidant and anti-inflammatory pathways, as well as enhanced microcirculation (35). Antioxidant-rich diets (e.g., vitamins C and E, carotenoids) can mitigate oxidative damage to cochlear cells, while anti-inflammatory diets may reduce inner ear inflammation (26, 49, 50). Prior studies suggest that higher oxidative balance scores (OBS) are inversely associated with the risk of hearing loss and tinnitus (11, 51). Similarly, elevated composite dietary antioxidant index (CDAI) levels correlate with reduced odds of hearing loss (12). Previous cross-sectional study of this cohort detected higher adherence to plant-based or healthy plant-based dietary indices correlates with a lower prevalence of hearing loss (35). Diets rich in anti-inflammatory and antioxidant components may reduce the risk of ARHL by up to 50%. Conversely, pro-inflammatory diets may increase the risk of sensorineural hearing loss (SNHL) (47). Excessive consumption of fats, and cholesterol has been linked to hearing loss, likely due to their association with cardiovascular diseases and impaired cochlear perfusion. Chronic high-fat diets may also alter gut microbiota, triggering systemic immune responses that compromise the blood-labyrinthine barrier (BLB) and promote cochlear inflammation. These findings highlighting the potential role of dietary interventions in prevention.

Plant-based DDS, emphasizing high intake of vegetables, fruits, whole grains, nuts, and legumes, provide abundant antioxidants and phytochemicals that protect against age-related damage. Such diets may also prevent vascular dysfunction by improving lipid profiles, endothelial function, and blood pressure, thereby enhancing cochlear blood flow. Additionally, healthy diets may reduce neuroinflammation and neurodegeneration in the central auditory pathway. Balanced diets promote vascular health, which is critical for maintaining inner ear microcirculation (52). Vegetable and fruit intake, particularly dark-colored varieties, leafy greens, berries, and citrus fruits, is associated with reduced risk of age-related hearing loss (ARHL), specifically high-frequency loss (18). Cohort studies indicate a 20–30% lower risk in high-intake groups relative to low-intake counterparts (26, 32). Legumes consumption correlates with favorable auditory outcomes within healthy dietary patterns, though dedicated studies remain limited. Legumes are consistently included as health-promoting components in diet quality assessments. Nuts consumption (≥2 servings/week) demonstrates an inverse association with hearing loss risk, putatively attributed to unsaturated fatty acids, vitamin E, and polyphenols (53). As a key constituent of the Mediterranean diet, nuts contribute to its established auditory benefits. Cereals confer protective effects compared to refined grains. High-glycemic-load diets elevate risk, whereas whole grain-induced stabilization of blood glucose levels supports auditory preservation (30). Fungi, containing ergothioneine and vitamin D, align with hearing-protective dietary principles when incorporated into plant-based diets. However, direct epidemiological evidence is currently lacking. Critically, protection primarily derives from synergistic interactions within plant-dominant dietary patterns (Mediterranean, DASH diets), as opposed to isolated foods or nutrients.

We observed variations in the associations between dietary diversity and hearing loss across gender, age groups and chronic diseases. The protective effects of plant-based dietary diversity were more pronounced in men aged 60–79, potentially attributable to higher intake of dietary antioxidants at younger ages, which may be more effective in preventing hearing loss. Hormonal decline associated with aging may also impact hearing, particularly through the reduction in estrogen levels among females (54, 55). The decline in estrogen levels in postmenopausal women contributes to increased oxidative stress. In our study, given the relatively high proportion of older adult women, the prolonged menopause period and lower sex hormone levels may obscure the independent protective effect of diet (56). These findings underscore the preventive potential of dietary interventions during the premenopausal period. Analysis of non-chronic disease subgroups in Japan’s NILS-LSA cohort revealed that the highest plant diversity group (≥6 species/day) had a 24% lower risk of hearing loss (57). In normotensive individuals, higher fruit and vegetable intake is inversely associated with hearing loss risk, while nut and legume consumption demonstrates significant protective effects (potentially mediated by magnesium and B vitamins). Furthermore, folate and vitamin C intake is negatively correlated with hearing loss risk among non-hypertensive women (26). While some studies suggest plant-based dietary diversity provides stronger protection in chronic disease populations, the relatively small proportion of such individuals in our study may introduce potential selection bias.

Our study possesses several strengths. First, we utilized a large, nationally representative sample of older adults in China, which enhances the generalizability of our findings. We made a prospective observation and conducted stratification analyses based on sex and age. Second, we employed DDS as a comprehensive measure of dietary quality, which avoids the limitations of focusing on specific nutrients or foods. Third, sensitivity analyses, which excluded participants who were unable to respond to questions due to hearing loss and those who developed hearing problems before age 40, confirmed the stability of our results. However, several limitations should be acknowledged. First, dietary data were self-reported, which may introduce memory bias and inaccuracies in reflecting actual food intake. Second, we lacked information on noise exposure, a critical confounding factor in hearing loss studies. Finally, despite adjusting for an extensive array of confounders, we cannot entirely exclude the possibility of residual or unmeasured confounding, given the inherent limitations of our observational study design.

## 5 Conclusion

In conclusion, our study provides evidence that higher dietary diversity, particularly in plant-based diets, is associated with a reduced risk of hearing loss in older adults. These findings underscore the importance of promoting dietary diversity as a potential strategy for preventing hearing loss. Future research should explore the underlying mechanisms in greater detail and investigate the potential benefits of dietary interventions in diverse populations.

## Data Availability

The datasets presented in this study can be found in online repositories. The names of the repository/repositories and accession number(s) can be found: https://doi.org/10.18170/DVN/WBO7LK.
