# Supplementary material for: The association between dietary diversity and hearing loss: results from a nationwide survey
Source: Front Nutr. 2025 Sep 8;12:1629685. doi: 10.3389/fnut.2025.1629685 (PMC12450950; doi:10.3389/fnut.2025.1629685)
Supplement: Supplementary file 1 [file Table_1.DOCX]

**Supplementary Table1** Subgroups analyses of the associations between DDS and hearing loss

| **Variables** | **DDS** | | | |
| --- | --- | --- | --- | --- |
|  | **Total** | **Animal-based** | **Protein-based** | **Plant-based** |
| **Gender** |  |  |  |  |
| Male |  |  |  |  |
| Q1 | Reference | Reference | Reference | Reference |
| Q2 | 1.064(0.873,1.298) | 1.058(0.879,1.273) | 0.951(0.790,1.145) | 0.905(0.758,1.080) |
| Q3 | 0.973(0.805,1.184) | 1.020(0.848,1.227) | 0.907(0.756,1.089) | 0.829(0.681,1.009) |
| Q4 | 0.818(0.667,1.002) | 0.762(0.538,1.079) | 0.771(0.587,1.012) | **0.751(0.567,0.995) *** |
| *P*-trend | 0.061 | 0.457 | 0.067 | **0.019** |
| Female |  |  |  |  |
| Q1 | Reference | Reference | Reference | Reference |
| Q2 | 1.145(0.949,1.382) | 1.008(0.843,1.205) | 1.071(0.897,1.280) | 0.845(0.710,1.006) |
| Q3 | 0.921(0.748,1.133) | 1.131(0.950,1.345) | 1.036 (0.864,1.243) | 0.998(0.826,1.206) |
| Q4 | 1.131(0.926,1.381) | 1.089(0.754,1.572) | 1.080(0.795,1.467) | 0.874(0.642,1.190) |
| *P*-trend | 0.782 | 0.418 | 0.559 | 0.670 |
| **Age group,year** |  |  |  |  |
| 60-79 |  |  |  |  |
| Q1 | Reference | Reference | Reference | Reference |
| Q2 | 1.205(0.983,1.478) | 1.110(0.920,1.339) | 1.106(0.915,1.337) | 0.898(0.745,1.081) |
| Q3 | 0.998(0.811,1.229) | 1.090(0.902,1.316) | 0.949(0.785,1.148) | 0.851(0.695,1.042) |
| Q4 | 0.911(0.743,1.117) | 0.859(0.620,1.188) | 0.887(0.685,1.148) | 0.806(0.622,1.061) |
| *P*-trend | 0.264 | 0.969 | 0.335 | 0.078 |
| ≥80 |  |  |  |  |
| Q1 | Reference | Reference | Reference | Reference |
| Q2 | 1.021(0.849,1.227) | 0.982(0.823,1.172) | 0.927(0.779,1.103) | **0.821(0.694,0.977) *** |
| Q3 | 0.890(0.731,1.083) | 1.052(0.887,1.248) | 0.968(0.812,1.155) | 0.922(0.766,1.109) |
| Q4 | 0.919(0.787,1.179) | 0.878(0.583,1.322) | 0.807(0.578,1.126) | **0.672(0.477,0.948) *** |
| *P*-trend | 0.447 | 0.802 | 0.322 | **0.064** |
| **Hypertension** |  |  |  |  |
| Yes |  |  |  |  |
| Q1 | Reference | Reference | Reference | Reference |
| Q2 | 1.220(0.945,1.575) | 1.189(0.938,1.507) | 1.068(0.845,1.351) | 0.943(0.749,1.187) |
| Q3 | 1.158(0.897,1.495) | 1.322(1.053,1.661) | 1.111(0.885,1.159) | 0.957(0.750,1.221) |
| Q4 | 1.042(0.812,1.338) | 1.034(0.677,1.580) | 0.915(0.641,1.307) | 0.799(0.558,1.145) |
| *P-*trend | 0.697 | 0.080 | 0.802 | 0.337 |
| No |  |  |  |  |
| Q1 | Reference | Reference | Reference | Reference |
| Q2 | 1.081(0.920,1.271) | 0.991(0.851,1.154) | 0.995(0.854,1.159) | **0.853(0.736,0.987)** * |
| Q3 | 0.880(0.742,1.044) | 0.988(0.849,1.149) | 0.912(0.780,1.066) | 0.878(0.744,1.035) |
| Q4 | 0.916(0.770,1.090) | 0.830(0.607,1.136) | 0.866(0.678,1.106) | 0.786(0.611,1.012) |
| *P*-trend | 0.140 | 0.502 | 0.150 | **0.043** |
| **Diabetes** |  |  |  |  |
| Yes |  |  |  |  |
| Q1 | Reference | Reference | Reference | Reference |
| Q2 | 1.113(0.535,2.315) | 0.963(0.492,1.885) | 1.257(0.681,2.320) | 0.864(0.429,1.737) |
| Q3 | 1.081(0.533,2.194) | 1.290(0.703,2.365) | 1.221(0.653,2.280) | 0.969(0.501,1.875) |
| Q4 | 1.094(0.592,2.024) | 1.056(0.463,2.408) | 1.080(0.497,2.346) | 0.994(0.449,2.201) |
| *P*-trend | 0.800 | 0.540 | 0.712 | 0.962 |
| No |  |  |  |  |
| Q1 | Reference | Reference | Reference | Reference |
| Q2 | 1.123(0.977,1.290 | 1.054(0.925,1.202) | 1.011(0.887,1.153) | 0.882(0.777,1.000) |
| Q3 | 0.957(0.828,1.106) | 1.074(0.944,1.222) | 0.969(0.849,1.106) | 0.906(0.788,1.042) |
| Q4 | 0.987(0.879,1.109) | 0.860(0.657,1.125) | 0.858(0.695,1.059) | **0.761(0.614,0.944)** * |
| *P*-trend | 0.294 | 0.783 | 0.245 | **0.021** |
| **Heart disease** |  |  |  |  |
| Yes |  |  |  |  |
| Q1 | Reference | Reference | Reference | Reference |
| Q2 | 1.083(0.697,1.683) | 1.020(0.683,1.521) | 1.236(0.838,1.822) | 1.025(0.691,1.520) |
| Q3 | 1.078(0.714,1.627) | 1.189(0.818,1.730) | 1.105(0.759,1.608) | 0.969(0.646,1.454) |
| Q4 | 1.000(0.671,1.491) | 0.983(0.546,1.768) | 0.992(0.594,1.657) | 1.041(0.612,1.772) |
| *P*-trend | 0.978 | 0.558 | 0.889 | 0.996 |
| No |  |  |  |  |
| Q1 | Reference | Reference | Reference | Reference |
| Q2 | 1.117(0.967,1.289) | 1.041(0.909,1.193) | 0.993(0.866,1.138) | **0.858(0.753,0.977)** * |
| Q3 | 0.943(0.810,1.097) | 1.064(0.930,1.218) | 0.955(0.831,1.096) | 0.904(0.782,1.045) |
| Q4 | 0.944(0.809,1.100) | 0.853(0.643,1.131) | 0.858(0.688,1.071) | **0.743(0.592,0.933)** * |
| \| *P*-trend \| 0.276 \| 0.873 \| 0.211 \| **0.019** \| \| --- \| --- \| --- \| --- \| --- \| | | | | |

Subgroups according to gender, age, hypertension, diabetes, heart disease;The results were based on Model 4 controlling for gender, age, household income, education level, marital status, pre-retirement occupation, physical activities, current smoking, current consuming alcohol, sleep quality, BMI, hypertension, diabetes, and heart disease; DDS, dietary diversity score; Q1-Q4: lowest quartile to the highest quartile.**P*<0.05.
